# Effects of hTERT immortalization on osteogenic and adipogenic differentiation of dental pulp stem cells

**DOI:** 10.1016/j.dib.2016.01.009

**Published:** 2016-01-14

**Authors:** El-Ayachi Ikbale, Sarita Goorha, Lawrence T. Reiter, Gustavo A. Miranda-Carboni

**Affiliations:** a Department of Medicine, University of Tennessee Health Science Center, Memphis, TN 38163, USA; b Department of Neurology, University of Tennessee Health Science Center, Memphis, TN 38163, USA; c Department Anatomy and Neurobiology, University of Tennessee Health Science Center, Memphis, TN 38163, USA; d Department of Pediatrics, University of Tennessee Health Science Center, Memphis, TN 38163, USA

**Keywords:** Stem cells, Osteogenic, Adipogenic, Immortalized, hTERT, DPSC

## Abstract

These data relate to the differentiation of human dental pulp stem cells (DPSC) and DPSC immortalized by constitutively expressing human telomerase reverse transcriptase (hTERT) through both osteogenic and adipogenic lineages (i.e. to make bone producing and fat producing cells from these dental pulp stem cells). The data augment another study to characterize immortalized DPSC for the study of neurogenetic “Characterization of neurons from immortalized dental pulp stem cells for the study of neurogenetic disorders” [1]. Two copies of one typical control cell line (technical replicates) were used in this study. The data represent the differentiation of primary DPSC into osteoblast cells approximately 60% more effectively than hTERT immortalized DPSC. Conversely, both primary and immortalized DPSC are poorly differentiated into adipocytes. The mRNA expression levels for both early and late adipogenic and osteogenic gene markers are shown.

**Specifications Table**

**Table:** 

Subject area	*Biology*
More specific subject area	*Stem cell biology, osteogenesis, adipogenesis*
Type of data	*Figure*
How data was acquired	*Microscopy imaging (Bright-field), qRT-PCR*
Data format	*Analyzed and annotated figure.*
Experimental factors	*Two human DPSC lines from (Urraca et al 2015): TP-023 and TP-023(I)*
Experimental features	*Both immortalized and non-immortalized DPSC at passage 5 were subjected to osteogenic and adipogenic stimulation and then stained with either Alizarin Red to detect calcium deposits and or Oil Red O to detect lipid in the culture. RNA was also extracted for qRT-PCR in undifferentiated vs differentiated cells.*
Data source location	*N/A*
Data accessibility	*Data is shown in this article*

Value of the data

• The effects of hTERT immortalization on the ability of DPSC to differentiate into osteocytes and adipocytes was previously unknown.
• These data may assist researchers in the decision to use hTERT immortalization of a cell line depending on the desired lineage.
• These data support the use of DPSC for the generation of adipocytes differentiation.

## 1 Data

### 1.1 Overview

The data shown are microscopy images (Bright-field) and qRT-PCR of Non-immortalized DPSC (NI-DPSC) and Immortalized DPSC (I-DPSC) at passage 5.

## 2 Experimental design, materials and methods

**Fig. 1:**
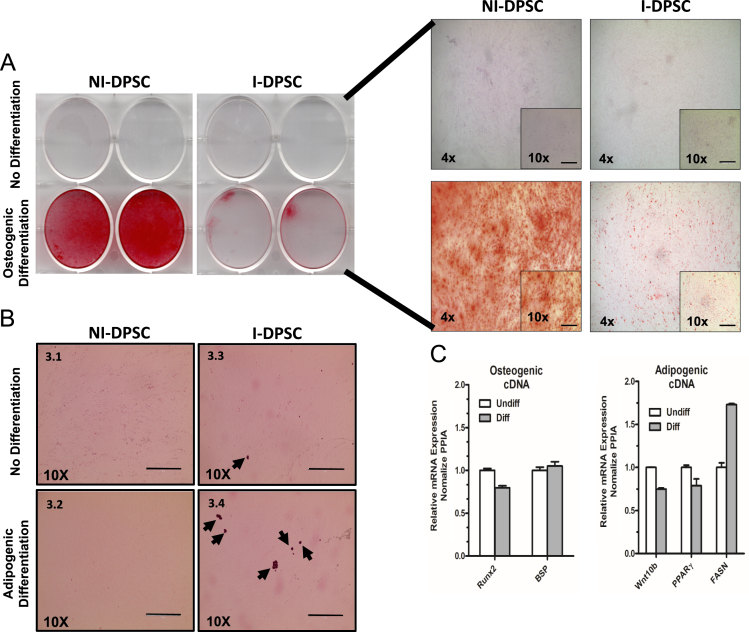
Non-immortalized DPSC (NI-DPSC) and Immortalized DPSC (I-DPSC) were used at passage 5. (A) Cells were stained with Alizarin Red after 21 days of differentiation and pictures show staining at 4× and 10× magnification. (B) Cells were stained with Oil Red O after 21 days of differentiation. Pictures show no difference between undifferentiated NI-DPSC and differentiated NI-DPSC. But very slight difference between undifferentiated I-DPSC and differentiated I-DPSC (black arrows indicate lipid droplets). (C) Gene expression of osteogenic (*RUNX2 and BSP*) and adipogenic (*WNT10B, PPAR*γ *and FASN*) markers. Relative mRNA expression is normalized with *PPIA* and standard deviation is indicated by error bars.

Adipogenic and osteogenic differentiation of DPSC was as previously described for bone [2] and for fat [3]. Briefly, the differentiation was conducted at passage 5 for both immortalized DPSC and primary DPSC each was grown for 21 days in Adipogenic media (Lonza) then fixed with Formalin 10% and 60% isopropanol and stained with Oil Red O, or in osteoblast differentiation media (DMEM 1×, 10% FBS supplemented with 50 μg/mL Ascorbic Acid-2-phosphate, 10 mM β-glycerophospahte, 10 nM dexamethasone, 10 nM 1,25-dihydroxyvitamin D3) then fixed with 50% ethanol and stained with Alizarin Red. For gene expression studies of osteogenic and adipogenic markers qRT-PCR was run in duplicate technical replicates and standard deviation calculated using Graph Pad software. Fig. 1

1. *RUNX2*: Forward: -TTT GCA CTG GGT CAT GTG TT-, Reverse: -TGG CTG CAT TGA AAA GAC TG-
2. *BSP*: Forward: -TGAA ACG AGT CAG CTG GATG-; Reverse: -TGA AAT TCA TGG CTG TGG AA-
3. *WNT10B*: Forward: -TGG GAT GTG TAG CCT TCT CC-; Reverse: -CCC AGC CAA AAG GAG TAT GA-
4. *PPARϒ*: Forward: -GTC GTG CAG GAG ATC ACA GA-; Reverse: -GGG CTC CAT AAA GT CAC CAA-
5. *FASN*: Forward: -TCC TGA GCA TGC TGA ACG AC-; Reverse: -AGC AGA TGA ACC AGA GCG G-
6. *PPIA*: Forward:-CAG ACA AGG TCC CAA AGA CAG-; Reverse: -TTG CCA TCC AAC CAC TCA GTC-

## 3 Human subjects

Teeth for the generation of DPSC were obtained through the Department of Pediatric Dentistry at the University of Tennessee Health Science Center (UTHSC). The UTHSC Institutional Review Board approved this study and informed consent was obtained from the parent or legal guardian of all participants.
